# Editorial: Reviews in metabolomics: Personalized and predictive medicine of infectious diseases

**DOI:** 10.3389/fmolb.2023.1178115

**Published:** 2023-03-20

**Authors:** Ilse Du Preez, Nguyen Phuoc Long

**Affiliations:** ^1^ Centre for Human Metabolomics, North-West University, Potchefstroom, South Africa; ^2^ Department of Pharmacology and PharmacoGenomics Research Center, Inje University College of Medicine, Busan, Republic of Korea

**Keywords:** metabolomics, infectious diseases, personalized medicine, tuberculosis, COVID-19

In the past few decades, metabolomics has become a valuable research tool, and its clinical applications are becoming more apparent. With this approach, the metabolic composition of a living system can be identified and quantitatively measured. The comprehensiveness of metabolomics data facilitates a more holistic view of human physiology and pathophysiology compared to typical serendipity-driven approaches. The resulting fingerprint represents the final downstream phenotype of genetic and transcription processes, and even subtle perturbations affecting this cascade will lead to detectable metabolome variations. These inter-individual perturbations can be inborn, such as gender or ethnicity, or acquired, in the case of diet or disease states, for example. By characterising these phenotypes, the underlying mechanism of the studied perturbation can be described better, and, in the case of diseases, can assist in the development of improved, and more personalised diagnostic and treatment approaches. The aim of this Research Topic was to specifically discuss the applications of metabolomics in personal medicine of infectious diseases.

Metabolomics is underpinned by highly advanced analytical technologies, each with its own advantages and challenges. Zhou and Zhong (2022), reviewed the application of one such instrument, liquid-chromatography mass spectrometry (LC-MS), for personalised medicine. From the extensive summary, it can be deduced that this approach is rapidly expanding, and has, in the past 2 years, led to the identification of metabolic fingerprints for various diseases, including hepatocellular carcinoma, breast cancer, and COVID-19, amongst others. The outcomes of these studies showed promise in the areas of molecular phenotyping, prognostics, diagnostics, and monitoring of disease progression. LC-MS can undeniably play a significant role in the future of precision medicine. However, processes and methods across laboratories should be standardised before its routine clinical implementation.

Although, in theory, metabolomics is a favourable future clinical tool, the reality is much more complex. The research applications of these methodologies are exponentially expanding; however, the practical implementation thereof still lacks. One way to overcome this, is to take one step back, and aim to better understand normal physiological processes, such as immunity. In accordance, Vivas-Garcia and Efeyan (2022), provided an overview of the molecular switches controlling the B-cell metabolism and fuel, and touched on the metabolic adaptions of B-cells during the different humoral response phases. They indicated that the integration between the metabolism and immunity is only surfacing and that a deeper understanding of this cross-talk is needed. Continued technological improvements, would, however, soon enable the analyses of single-cells, which would facilitate high throughput studies of, for example, B-cell metabolism.

In a similar fashion, Guo et al. (2021) reviewed the physiological and pathological functions of angiotensin-converting enzyme 2 (ACE2). To elaborate on this Research Topic, the impact of ACE2 on the gastrointestinal system through mTOR (affecting tryptophan absorption), and the renin-angiotensin system (RAS) is discussed. Details of the regulating mechanism of this enzyme show that it acts as a binding protein for severe acute respiratory syndrome coronavirus-2 (SARS-CoV-2) cell entry, although future studies, focussing on the different organs involved, are recommended. Further investigations could lead to the identification of targets for treatment efficiency monitoring in COVID-19 patients.

Many other aspects of COVID-19 have also been investigated using an array of metabolomics techniques, as summarised by Bruzzone et al. (2023). By the time of publication, an astonishing 90 original contributions were made covering this field, with a consensus on the metabolic signature of the acute phase of the disease. Other applications of metabolomics include identifying COVID-19 severity biomarkers, drug-induced metabolome variations and the metabolic recovery of patients. A significant finding is long-term abnormalities of the lipoprotein metabolism seen in these patients, potentially explaining the post-infection risk of cardiovascular episodes. This review indicates the broad application of metabolomics toward disease characterisation, and the impact these studies can have in a short time, when resources and funding are readily available.

In contrast to the COVID-19 pandemic, which had a sudden onset and rapid spread, tuberculosis (TB) has been classified as an epidemic in various countries for decades. Accordingly, since the initiation of the metabolomics research field, it has been applied to study various aspects of this disease, using a variety of sample matrixes, as reviewed by Yu et al. (2023). Biomarkers identified mostly represent those reflecting the host response to the disease, including active, latent, and extrapulmonary TB. Metabolic signatures for therapeutic monitoring and drug toxicity have also been identified from urine, blood, and cerebrospinal fluid. In addition to the host metabolome, the causing pathogen, *Mycobacterium tuberculosis*, has also been extensively studied using different metabolomics techniques. The outcomes of these studies provided a better understanding of host-pathogen interaction and drug resistance.

This compilation of reviews indicates the contribution that metabolomics can make towards a better understanding and description of various infectious diseases, ranging from basic biology to clinical applications. It should be realised; however, that metabolomics tends to be a hypothesis-generating domain, and the identified biomarkers and/or mechanisms should be validated before implementation as personalised medicine approaches. Finally, the integration of metabolomics into the multi-omics “eco-system” is necessary to bridge the gap between genome and phenome.

